# Current inventory and changes of the input/output balance of trace elements in farmland across China

**DOI:** 10.1371/journal.pone.0199460

**Published:** 2018-06-25

**Authors:** Runxiang Ni, Yibing Ma

**Affiliations:** Institute of Agricultural Resources and Regional Planning, Chinese Academy of Agricultural Sciences, Beijing, China; Sun Yat-Sen University, CHINA

## Abstract

The inventory and input/output balance of trace elements in farmland play an important role in risk assessment and soil management, but there is little information about nationwide changes of the input/output balance of trace elements in farmland in China. In the present study, the inventory of trace element inputs to farmland was updated based on the dataset from the literature published during 2006–2015, and changes of the input/output balance were investigated. Compared with 1999–2006, net inputs of Cr, Ni, and Zn increased by 52.9%, 59.7%, and 20.6%, respectively. The increases in fossil fuel derived energy consumption, industrial manufacture, municipal solid waste incineration, and transportation were the predominant contributors to these increases. Net inputs of Cd, Cu, and Hg decreased dramatically by 46.7%, 25.2%, and 50.4%, respectively. The decreases are due to the strict management of feed additives, fertilizers, and emissions of atmospheric pollutants. Net inputs of As and Pb still remained relatively stable. These results demonstrated that better achievements have been gained by administration of air, water and soil in China. Regulation of atmospheric emission for Cr, Ni, and Zn was recommended as atmospheric deposition was the predominant source for increases of Cr, Ni, and Zn inputs to farmland across China.

## 1. Introduction

The input/output balance of trace elements in farmland is a key issue in soil contamination and prevention. Furthermore, it is also of great importance to the risk assessment of soil contamination and management [1]. Generally, the concentrations of trace elements in soil are maintained in a dynamic input/output balance. The potential adverse effects of harmful element input to farmland soil can be eliminated by soil self-purification, when such inputs are small. However, a well-balanced agro-ecosystem can be easily disrupted when excess amounts of harmful elements are input to farmland soil. An input/output imbalance of trace elements could result in an accumulation of trace elements in farmland soil, and then lead to soil contamination. Harmful elements in a polluted soil could accumulate in crops to levels that threaten human health. An input/output imbalance of trace elements, especially for heavy metals, could cause huge losses to agriculture globally, as well as China [2]. It may take hundreds of years to recover this imbalance by soil self-purification, and even with human intervention the available time and finances may limit contaminant removal. A detailed quantitative analysis of the trace element balance in soils would enable managers to better understand soil quality status and the tendency, and effective policies could then be established to protect the soil resources.

The studies on input/output balance of trace elements in farmland have been reported in several countries. Among all possible sources of trace elements, atmospheric deposition is the predominant contributor to most of the inputs to farmland, especially in more industrial countries such as China [3] and the UK [4]. In contrast, animal manures, mineral fertilizers, and pesticides are the predominant sources of trace elements in France [5]. The input/output balances of trace elements in regional farmland in China (Hainan [6], Heilongjiang [7], and the Yangtze River Delta [8]) have been analyzed by Chinese scientists. Luo et al. [3] compiled a nationwide inventory of trace element inputs to agricultural soils based on published literature (1999–2006) and national statistics. They reported that cadmium (Cd) is the most risk and first priority pollutant in farmland across China. The nationwide trace element input/output balance sheet plays a significant role in soil heavy metal pollution prevention and control in China. Soil contamination with heavy metals has been paid more and more attention in China. Environmental protection and food safety requirements for Chinese residents are increasingly strict, and much work in this area has been undertaken by the Chinese government in recent years. For example, the Safety Specification of Feed Additives was released in 2009 [9], atmospheric pollutant emission standards were formulated and updated in 2010, and sewage irrigation was totally banned in 2013 [10]. These policies will influence the trace element input/output balance in farmland across China; however, few studies have focused on these nationwide changes.

In the present study, a trace element input/output balance sheet in farmland across China was compiled based on dataset from the literature published during 2006–2015 and national statistics [11–13] Input sources of atmospheric deposition, livestock manures, fertilizers, agrochemicals, irrigation and sewage sludge, and crop harvesting and straw removal outputs were included in this new inventory. The status, changes, and causes for the trace element input/output in farmland in China were analyzed. The results of this study will be helpful to understand the sources, status, and trend of trace elements in farmland and assist administrative and legislative agencies, as well as managers, to prevent the contamination of trace elements in farmland and to remediate contaminated land in China.

## 2. Materials and methods

The different input sources of trace elements, such as atmospheric deposition, livestock manures, fertilizers, pesticides, irrigation water, and sewage sludge, were selected in the present study. As trace element outputs, crop harvesting and straw removal were only selected because related data were used to estimate trace element leaching from farmland are hardly available. Leaching has been identified as the main output pathway for trace elements in agricultural soil in areas like Yangtze River delta and southern Song-nen Plain in China [7, 8]. However, compared with the input fluxes, the estimated output fluxes of trace elements are relatively small [6–8] and the data are scarce. Therefore, the trace element amounts of leaching from soils can be ignored. The calculation procedures for the trace element input/output balance in the present study referred mainly to the publications by Luo et al. [3], Nicholson et al. [4], and Belon et al. [5]. The trace elements in the present study included arsenic (As), Cd, chromium (Cr), copper (Cu), mercury (Hg), nickel (Ni), lead (Pb), and zinc (Zn).

### 2.1 Estimation of inputs

#### 2.1.1 Atmospheric deposition

The trace element inputs to farmland from atmospheric deposition were obtained by multiplying the farmland area in China (1.35×10^8^ hectares [11]) by the averaged (as geometric means) atmospheric deposition fluxes of trace elements (Table A in S1 Supporting Information). Sampling time or duration, site characteristics, as well as the representativeness of samples, were taken into consideration when averages of atmospheric deposition fluxes of trace elements were estimated (Table A in S1 Supporting Information). In order to compare the atmospheric deposition of trace elements, the atmospheric deposition fluxes of trace elements in other countries or districts were also listed in Table A in S1 Supporting Information.

#### 2.1.2 Livestock manures

The trace element inputs to farmland from livestock manures were calculated as follows:

$$A_{i}=f\sum_{j}N_{j}P_{j}(1-f_{wj})C_{ij}$$ (1)

where *A*_*i*_ is the input of a trace element (*i*) to farmland from livestock manures; *f* is the agricultural application ratio of livestock manures in China, which varied from 31% to 56% and was 42% as a geometric mean (Table B in S1 Supporting Information); *N*_*j*_ is the number of livestock (*j*) raised annually [11]; *P*_*j*_ is the excretion parameter of livestock, which for different livestock were listed in Table C in S1 Supporting Information; *f*_*wj*_ is the water content in livestock manures, which for different livestock manures were listed in Table D in S1 Supporting Information; and *C*_*ij*_ is the trace element concentration in livestock manures (dry weight) in China, which for different livestock manures were listed in Table E in S1 Supporting Information. The excretion parameters of the livestock referred to Wang et al. [14], the number of animals raised annually referred to population of animals in 2015 [11], water contents and trace element concentrations in livestock manures, and the agricultural application ratio were updated based on literature published during 2006–2015 and national statistics (Tables C-E in S1 Supporting Information).

#### 2.1.3 Fertilizers and pesticides

The trace element inputs to farmland from fertilizers were calculated from the average concentration of trace elements in fertilizers, which is listed in Table F in S1 Supporting Information and the total amount of fertilizers consumed in 2015 [11]. Inputs of Cu and Zn to farmland from pesticides were estimated from statistics published by The National Agro-Tech Extension and Service Center; The consumptions of Cu sulfate, Cu hydroxide, and ethylenebisdithiocarbomate were all more than 1,500 tons in 2015 in China [15]; therefore, Cu and Zn inputs to farmland from pesticides were at least 1,580 and 355 tons, respectively.

#### 2.1.4 Irrigation water

The trace element inputs to farmland from irrigation water were estimated from the average concentrations of trace elements in irrigation water (Table G in S1 Supporting Information), irrigation area (6.587×10^7^ hectares [11]), and gross irrigation quota (5910 m^3^/hectare [16]).

#### 2.1.5 Sewage sludge

Agricultural uses of untreated sewage sludge have been banned in China from 2013 [10]. However, the amount of treated sewage sludge used in agricultural production in China is unavailable. Considering sewage sludge has been used in agricultural production for several years before 2013, the trace element input to farmland from sewage sludge estimated by Luo et al. [3] was adopted in the present study. The trace element input to farmland from sewage sludge was estimated from average concentrations of trace elements in sewage sludge [17] and total quantity of dry sewage sludge applied to farmland [18].

### 2.2 Estimation of outputs

The trace element outputs from farmland by crop harvesting were estimated from the yield of major crop products and average concentrations of trace elements in crops (Tables H-M in S1 Supporting Information). Compared with Luo et al. [3], not only the major food crop grains (rice, wheat, and maize), but also straws and other food crops, as well as oil-bearing crops, cotton, sugarcane, sugar beet, tobacco, tea, fruits, and vegetables were taken into consideration in the estimation of trace element outputs by crop harvesting.

The trace element outputs from farmland by straw removal in China were calculated as follows:

$$O_{i}=\sum_{j}O_{ij}=\sum_{j}W_{pj}\times S_{Gj}\times f_{j}\times C_{pij}\times \delta_{ij}$$ (2)

where *O*_*i*_ is the output of the trace element (*i*) from agricultural soil; *O*_*ij*_ is the trace element (*i*) output from agricultural soil by crop (*j*) straw removal; *W*_*pj*_ is the crop yield [11]; *f*_*j*_ is the rate of crop (*j*) straw removal from agricultural soil (31.9% [19]); *C*_*pij*_ is the trace element (*i*) concentration in crop (*j*) (Table H in S1 Supporting Information); *S*_*Gj*_ is the ratio of the yield of straw/grain (Table N in S1 Supporting Information); and *δ*_*ij*_ is the ratio of the trace element concentration in straw/grain (Table N in S1 Supporting Information). Because the trace element concentrations in other crop straws were unavailable and rice, wheat, and maize straw account for a large proportion of the total crop straw in China, only trace element outputs by rice, wheat, and maize straw removal were estimated in the present study.

## 3. Results and discussion

### 3.1 Changes of the trace element input/output balance

The status and changes of the trace element input/output balance in farmland are listed in Table 1. Total trace element inputs could be ranked as follows: Zn > Cu > Pb > Cr > Ni > As > Cd > Hg, which was in agreement with characteristics of the UK [4] and France [5]. The contributions of different sources to total inputs are listed in Table 2, which vary greatly for different elements. Atmospheric deposition was the major source, accounting for 54.8%–94.5% of the total input during 2006–2015 in China, except Cu (31.9%). Copper inputs were mostly due to livestock manures (63.4%). Zinc inputs were linked to both atmospheric deposition (54.8%) and livestock manures (43.8%). These were very different from France [5], where livestock manures, mineral fertilizer, and pesticides are the predominant sources in agricultural soils.

**Table 1:** Changes of the trace element input/output balance in farmland across China (tons/year).

		As	Cd	Cr	Cu	Hg	Ni	Pb	Zn
Atmospheric deposition	1999–2006	3451	493	7392	13145	174	7092	24658	78973
	2006–2015	4552	553	21783	15606	124	10908	25906	98414
Livestock manures	1999–2006	1412	778	6113	49229	23	2643	2594	95668
	2006–2015	1001	145	2851	31049	13	1581	1219	78599
Fertilizers	1999–2006	835	113	3429	2741	87	504	1565	7874
	2006–2015	109	16	618	505	3	1114	211	1489
Pesticides	1999–2006				5000				125
	2006–2015				1580				355
Irrigation water	1999–2006	219	30	51	1486	1.3	237	183	4432
	2006–2015	5.2	0.5	9.4	19	0.1	7.2	7.8	53
Sewage sludge	1999–2006	7.4	1.4	85	224	1.3	36	60	669
	2006–2015	As same as that between 1999–2006							
Total input	1999–2006	5925	1417	17071	71824	286	10512	29061	187741
	2006–2015	5674	717	25346	48983	141	13646	27404	179579
Total output	1999–2006	192	178	1038	12158	18.2	2432	208	60792
	2006–2015	286	57	829	4340	8.0	743	174	26539
Net input	1999–2006	5733	1239	16033	59666	268	8080	28853	126949
	2006–2015	5388	660	24517	44642	133	12903	27230	153040

Notes: Because the amount of sewage sludge for agricultural use is unavailable, the trace element input to farmland from sewage sludge estimated by Luo et al. [3] was adopted in the present study.

**Table 2:** The contribution of different sources for trace elements to total inputs in farmland across China (%).

		As	Cd	Cr	Cu	Hg	Ni	Pb	Zn
Atmospheric deposition	1999–2006	58.2	34.8	43.3	18.3	60.8	67.5	84.8	42.1
	2006–2015	80.2	77.1	85.9	31.9	87.9	79.9	94.5	54.8
Livestock manures	1999–2006	23.8	54.9	35.8	68.5	8.0	25.1	8.93	51.0
	2006–2015	17.6	20.2	11.2	63.4	9.2	11.6	4.45	43.8
Fertilizers	1999–2006	14.1	7.97	20.1	3.82	30.4	4.79	5.39	4.19
	2006–2015	1.92	2.23	2.44	1.03	2.13	8.16	0.77	0.83
Pesticides	1999–2006				6.96				0.07
	2006–2015				3.23				0.20
Irrigation water	1999–2006	3.70	2.12	0.30	2.07	0.45	2.25	0.63	2.36
	2006–2015	0.09	0.07	0.04	0.04	0.07	0.05	0.03	0.03
Sewage sludge	1999–2006	0.12	0.10	0.50	0.31	0.45	0.34	0.21	0.36
	2006–2015	As same as that between 1999–2006							

Notes: the data in Table 2 were calculated from Table 1.

Total trace element inputs during 1999–2006 in China were selected for comparison [3], so that the changes of the total trace element inputs were calculated by the input of 2006–2015 minus the input of 1999–2006 then divided by the input of 1999–2006, which are expressed as percentages and listed in Table 3. The results showed that total As, Pb, and Zn inputs decreased slightly as 4.24%, 5.70%, and 4.35%, respectively; total Cd, Cu, and Hg inputs decreased sharply as 49.4%, 31.8%, and 50.7%, respectively; but Cr and Ni inputs increased remarkably as 48.5% and 29.8%, respectively (Table 3). When contribution was expressed as a percentage, livestock manures accounted for most of the decreases for Cd (84.1%) and Cu (72.2%). Fertilizers were the biggest contributor (57.8%) to the decrease in the Hg input, followed by atmospheric deposition (34.5%). Only atmospheric deposition contributed to Cr increase, and atmospheric deposition was also the predominant source of total Ni increase and account for 81.7%. The causes for the changes of the total trace element inputs were discussed in the following sections.

**Table 3:** The change (%) of trace element inputs to farmland from 1999–2006 to 2006–2015.

	As	Cd	Cr	Cu	Hg	Ni	Pb	Zn
Atmospheric deposition	31.9	12.2	194.7	18.7	-28.7	53.8	5.06	24.6
Livestock manures	-29.1	-81.4	-53.4	-36.9	-43.5	-40.2	-53.0	-17.8
Fertilizers	-86.9	-85.8	-82.0	-81.6	-96.6	121.0	-86.5	-81.1
Pesticides				-68.4				355
Irrigation water	-97.6	-98.3	-81.6	-98.7	-92.3	-97.0	-95.7	-98.8
Total input	-4.24	-49.4	48.5	-31.8	-50.7	29.8	-5.70	-4.35
Net input	-6.02	-46.7	52.9	-25.2	-50.4	59.7	-5.63	20.6

Notes: the data in Table 3 were calculated from Table 1 by the input of 2006–2015 minus the input of 1999–2006 then divided by the input of 1999–2006.

The trace element output from farmland by crop harvesting or straw removal varied markedly among elements and was influenced by trace element transfer factors and weight variations of grain and crop straw (Table N in S1 Supporting Information). Detailed information of trace element outputs by crop harvesting and straw removal can be found in Table O in S1 Supporting Information. The outputs of As, Cd, Cr, Hg, Ni, and Pb were mainly due to straw removal, while Cu and Zn outputs were mainly due to crop harvesting. In terms of crop harvesting, the main food crops (rice, wheat, and maize) and vegetables were the main output and accounted for 81.6% of the total (Table O in S1 Supporting Information). In terms of the different trace element outputs by crop harvesting, most of As, Cr, Cu, Ni, and Zn were output by the harvesting of the main food crops, while Cd, Hg, and Pb were output by vegetables (Table O in S1 Supporting Information). The contributions of tobacco to the Cd output and oil-bearing crops to the Ni output were second only to the contribution from the main food crops (Table O in S1 Supporting Information). In terms of straw removal, rice straw accounted for most of the As, Cu, Hg, Ni, and Zn output, maize straw accounted for most of the Cd and Cr output, and wheat straw accounted for most of the Pb output (Table O in S1 Supporting Information). Although the major food crop straws and more food crops, such as vegetables and oil-bearing crops, were taken into account for trace element outputs (see the section of Materials and methods), the outputs of all trace elements, except As, decreased by from 16.4% to 69.4%, while the output of As increased by 49.0%, which may be mainly due to the limited samples used during 1999–2006 [3].

A trace element input/output balance in farmland across China was calculated by subtracting the total output from the total input. Compared with the total net input during 1999–2006 [3], the net As and Pb inputs decreased slightly by 6.02% and 5.63%, respectively, the net Cd, Cu, and Hg inputs dropped by a relatively high amount (46.7%, 25.2%, and 50.3%, respectively), but the net Cr, Ni, and Zn inputs increased (52.9%, 59.7 and 20.6%, respectively) (Table 3). The results will supply the evidence for the effects of recent action plans for prevention and control of soil, water, and air pollution by Chinese government. However, more attention should be paid to Cr, Ni and Zn because they still increased when comparing with the total net inputs during 1999–2006 [3].

### 3.2 Atmospheric deposition

The changes of the trace element inputs to farmland from atmospheric deposition varied greatly among the different elements (Table 1). Considering the contribution of the growth in farmland area (about 10.6%), the changes of atmospheric deposition for Cd (12.2%), Cu (18.7%), and Pb (5.06%) were relatively small. However, there was a large decline (28.7%) in the Hg atmospheric deposition, while there was a relatively large increase in the As, Cr, Ni, and Zn atmospheric deposition (24.6%–194.7%) (Table 3). The causes for these changes were the variations in the atmospheric deposition fluxes (Table 4). Compared with the statistics for the period of 1999–2006 [3], the Cd, Cu, and Pb fluxes changed little, and the As, Cr, Cu, Ni, and Zn fluxes increased to different degrees. The largest increase was observed for Cr, with a flux 2.65 times of the value during 1999–2006. The Hg flux dropped dramatically (Table 4). However, compared with the atmospheric deposition fluxes in the overseas countries or districts, the atmospheric deposition fluxes of trace elements were still high in China, which ranged from 1.5 to 5.7 folds for Cd, Cu, Hg, Ni, Pb, and Zn; and from 11.2 to 14.2 folds for As and Cr. More details can be found in Table A in S1 Supporting Information.

**Table 4:** Atmospheric deposition flux of trace elements in China and overseas countries or districts (mg/m^2^/year).

	As	Cd	Cr	Cu	Hg	Ni	Pb	Zn
China (2006–2015)^a^	3.37	0.41	16.1	11.6	0.092	8.08	19.2	72.9
China (1999–2006)^b^	2.8	0.40	6.1	10.8	0.14	5.8	20.2	64.7
Overseas average^c^	0.30	0.18	1.14	4.46	0.032	1.41	3.88	50.24

^a^The present study.

^b^Luo et al. (2009) [3].

^c^Details are list in Table A in S1 Supporting Information.

**Fig 1:**
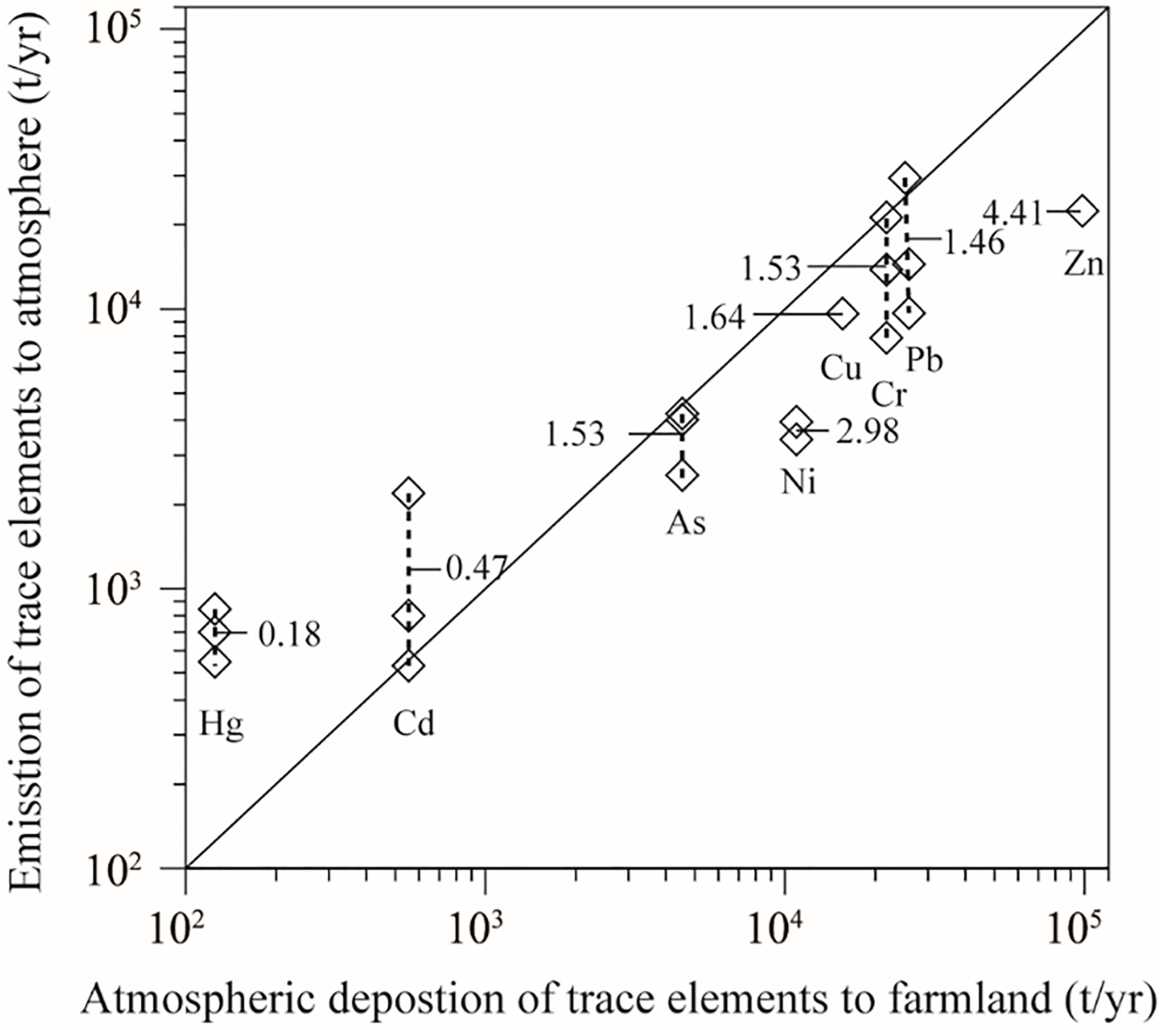
Comparison of atmospheric deposition of trace elements to farmland and emission of trace elements to atmosphere (ratios in the picture were calculated by input/emission).

The increases in fossil fuel derived energy consumption, industrial manufacture, municipal solid waste incineration, and transportation were the main sources of atmospheric trace elements in China [20], and atmospheric deposition was the main process that removed trace elements from the air [21, 22]. Estimates of the trace elements emitted from anthropogenic sources in recent years in China were used to validate the trace element atmospheric deposition to farmland which was estimated in the present study (Fig 1). The results showed that As, Cd, Cr, and Pb inputs to farmland from atmospheric deposition were in the range of estimated emissions, while Hg inputs to farmland from atmospheric deposition were much lower than emissions, which probably because of overestimation of Hg emission factors [23]. However, Cu, Ni, and Zn inputs to farmland from atmospheric deposition were much higher than emissions, which is probably because of lower estimation of their emissions due to the uncertainties of dynamic emissions and the assumptions in estimating emissions as indicated by Tian et al. [20].

The dramatic decrease in the Hg flux can be attributed to the strict atmospheric Hg emission management in China since 2010 [24]. There have been remarkable reductions in atmospheric Hg emissions in China due to technical upgrades and the installation of pollution control equipments [25–27]. As predicted by Cheng et al. [24], atmospheric emissions of Hg were reduced by 38% after new emission standards (GB 13223–2011, Emission standard of air pollutants for the thermal power plants [28]) for thermal power plants were introduced, which is in accordance with the observed reduction (34.1%) in the Hg flux (Table 4).

Cheng et al. [29] found that atmospheric emissions of Cr were consistent with economic development and energy consumption in China. Statistical data [13] showed that the Gross Domestic Product and energy consumption of China in 2015 were 3.12 and 1.5 times greater than those in 2006, which was similar to the growth rate of the Cr flux (1.65 times). The annual growth rate of the Ni flux (3.93%) was consistent with the average annual growth rate of Ni emissions (about 4%) estimated by Tian et al. [30] and slightly lower than the annual growth rate of coal and raw oil consumption (5.6% and 6.8%, respectively). Because anthropogenic Ni and Cr emissions to air in China have increased, the growth of Cr and Ni inputs to farmland estimated in the present study were reasonable. National statistics confirmed that coal and raw oil consumption, the yield of nonferrous metals, cement, plate glass, and pig iron, the amount of private automobiles in use, and municipal solid waste incineration all increased by 55.7 to 440% [13]. This has inevitably led to increases in trace element emissions and inputs to farmland.

It is notable that there are large spatial variations for atmospheric deposition of trace elements. The results (Table A in S1 Supporting Information showed that the atmospheric deposition flux of trace elements in China varied from 21 to 430 times for different trace elements, which suggested that the numbers and distributions of samples should be considered for a small scale or a regional assessments in order to decrease the uncertainties.

### 3.3 Livestock manures

**Fig 2:**
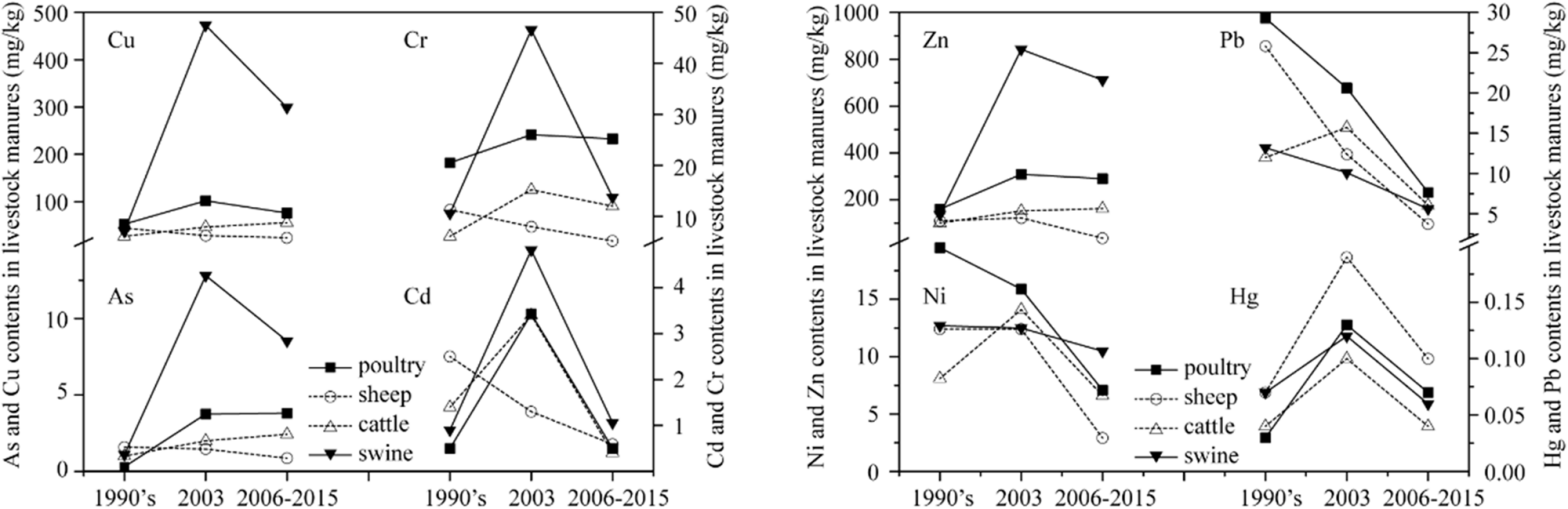
Changes of the trace element concentrations in livestock manures in China.

Trace element inputs from livestock manures to farmland declined over a large range from 17.8 to 81.4% (Table 3). The amounts of livestock manures used for agricultural production and the trace element concentrations in livestock manures are possible reasons for this decline. National statistics showed that the population of large livestock were reduced by 8.8%–22.4% during 2006–2015, but the agricultural application rate of livestock manures increased from 30% to 42%, which led to an increase of 29.7% in the total livestock manure inputs to farmland (Table C in S1 Supporting Information) Therefore, the trace element concentrations in livestock manures were the predominant factor controlling these declines. The trace element concentrations in livestock manures in China increased strongly from 1999–2010, and most of increase occurred before 2003, which reflects extensive use of feed additives before 2003 [31]. Compared with the livestock manures in China in 2003 [3], the trace element concentrations in livestock manures in 2006–2015 decreased dramatically by 15.5%-88.2%, except Cr and Zn concentrations in poultry decreased slightly by 3.2–6.2% and As, Cu, and Zn in cattle manures and As in poultry manures increased by 1.3%–21.5% (Fig 2).

The concentrations of Cr, Cu, and Zn in livestock manures are directly related to the feed additives used. The observed decreases of trace element concentrations in livestock manures can be attributed to the strict management of feed additives since 2009. The Safety Specification of Feed Additives was released by the Ministry of Agriculture of the People’s Republic of China (MOA) in 2009 [9], and a feed additives administration program was conducted immediately. Reports from the MOA and researchers have shown that the misuse and overuse of feed additives in some areas of China have been restrained. Among all trace elements, Cd is of special concern. Monitoring data from the MOA shows that Cd concentrations in feed throughout the country have decreased annually during the period of 2008–2013, and the Cd levels in feed after 2013 were lower than its limit [32, 33]. The trace elements of Cd, Ni, Hg, and Pb are prohibited in livestock breeding in China. Impurities in feed or additives provide a route for these trace elements to enter the feed-livestock-manure system. Arsanilic acid, also known as aminophenyl arsenic acid, and roxarsone are the main organic arsenic compounds used in animal drug which were also strictly restricted in China since 2005 [34]. Therefore, the concentrations of As, Cd, Ni, Hg, and Pb in livestock manures were much lower than those of Zn, Cu, and Cr. Although changes of the concentrations of the trace elements of As, Cd, Cr, Ni, and Hg in feed in China were unavailable, we can assume that the strict management of feed additives has prevented these trace elements from entering the feed-animal-manures system, leading to a decrease in their concentrations in manures.

### 3.4 Fertilizers

The percentage of fertilizer input fluxes for all elements ranged from 1% to 9% in Yangtze River delta, which was much lower than irrigation water ranged from 13% to 71% [8]. While, Cr and Cd inputs to French agricultural soils were mostly due to mineral fertilizers [5]. Phosphate and compound fertilizers are the predominant contributors to the input of trace elements to farmland from fertilizers, and account for 66.5%–94.8% of the total trace element inputs from fertilizers (Table F in S1 Supporting Information). Compared with 1999–2006, the input of all trace elements to farmland from fertilizers all dropped, except for Ni (Table 1). Variations in the total fertilizer use and trace element concentrations in fertilizers are possible reasons for this. National statistics showed that the total consumption of nitrogen, phosphate, potash, and compound fertilizers increased by 4.4%, 9.6%, 26.0%, and 57.0% during 2006–2015, respectively (Table F in S1 Supporting Information). The trace element concentrations in fertilizers were the predominant reason for these changes. Trace element concentrations in fertilizers were lower than those in 2003 [3], except for Cu and Pb in nitrogen fertilizers, Ni in phosphate and compound fertilizers, and Cd in potassium fertilizers. Although the Cd, Cu, and Pb concentrations in nitrogen and potassium fertilizers increased, they were still much lower than the concentrations in phosphate and compound fertilizers; therefore, their contributions to changes of the input of trace elements were negligible.

Strict fertilizer management was responsible for the decrease in trace element concentrations in fertilizers in China. The Fertilizer Registration and Management regulation was released in 2000 by the MOA [35]. Fertilizer production technologies have been updated and fertilizers in China have been purified. Phosphate and its raw materials have attracted much attention. There have been advances in phosphate purification in China [36]. Phosphoric acid is one of the main raw materials in phosphate and compound fertilizers. Phosphoric acid production in a blast furnace process was initiated in 2009, and phosphates produced via this new process contain much lower trace elements than those produced by the traditional wet-process [37].

Nickel inputs to farmland from fertilizers increased by 121.0%. There were two possible reasons for this. The Ni concentrations in nitrogen and potassium fertilizers were unavailable in 2003 [3]; therefore, Ni inputs to farmland from fertilizers were underestimated. The Ni concentrations in phosphate and compound fertilizers were much higher than those in 2003 [3]. Nickel compounds are important catalysts in fertilizer production and Ni may remain in the final products [38]. Additionally, there are no limits for Cu, Ni, and Zn in Chinese fertilizer standards (GB/T 23349–2009 [39]) because they are also essential or beneficial nutrients; therefore, these elements have been assessed barely during fertilizer production.

### 3.5 Irrigation water and sewage sludge

Generally, the input fluxes from irrigation water for the trace elements were much smaller than those from other input pathways, such as atmospheric deposition and fertilization [7], except areas where irrigation water was polluted by industrial or domestic sewage disposals [8]. Sewage irrigation has caused huge economic losses to agriculture in China, totally monetary equivalent to 11.54 × 108–14.36 × 10^8^ US dollars during 2010–2013 [40]. The adverse effects of sewage irrigation have been noted by the Chinese government, and sewage irrigation was totally banned in China in 2013 [10]. Surface and underground water are now the main sources of irrigation water in China. The average concentrations of trace elements in irrigation water estimated in the present study (Table G in S1 Supporting Information) were much lower than the relevant limits. Therefore, trace element inputs to farmland from irrigation water have declined dramatically by 81.6%–98.8% (Table 3). However, there is wide spread contamination of agricultural land which is caused by surface runoff from mining areas and industrial facilities and by wastewater irrigation.

Sewage sludge from municipal and industrial sewage contains large amounts of organic matter and nutrients of nitrogen, phosphorus and potassium and therefore sewage sludge is used in agricultural production to improve soil quality [41]. Statistics have shown that the amount of sewage sludge generated and land use in China have increased by 33.0% and 58.5% of those in 2011, respectively, during 2011–2015 [42]. Zhang et al. [43] found that about 25% of Ni concentration and 10% of Cd, Cr, Cu, Hg, Ni, and Zn concentrations in the sewage sludge were higher than related standards. The comprehensive utilization rate of sewage sludge is expected to 90% in city areas in China [44]. Considering the trace element concentrations in sewage sludge in China [43], we suggest that more attention should be paid to sewage sludge agricultural use in China.

It is necessary to indicate that this current inventory of trace elements in farmland in China is based on dataset about the sources of trace elements reported in literatures and the scale is nationwide. Because of the spatial variation of contaminant sources and distribution, there are some areas of serious soil pollution in China with relevance to anthropogenic activities [45, 46], so that more attention should be paid when this inventory applies to a regional or individual field scale.

## 4. Conclusion

Current inventory and changes of the trace elements input/output balance in farmland in China nationwide were analyzed in the present study. From 1999–2006 to 2006–2015, net inputs of As and Pb experienced smaller declines of 6.02% and 5.63%, respectively; and net inputs of Cd, Cu, and Hg dropped dramatically as 46.7%, 25.2% and 50.4%, respectively. These results demonstrated that better achievements have been gained by administration of air, water and soil in China and proved that strict management of atmospheric pollutants emission, feed additives use, and fertilizers production and application were effective ways to prevent farmland from contamination. What calls special attention is that net inputs of Cr, Ni, and Zn increased by 52.9%, 59.7% and 20.6%, respectively. The increasing amount of fossil energy consumption, industrial manufacture, municipal solid waste incineration, and transportation were the predominant reasons for these increases. Therefore, we recommend that the atmospheric emission standards of pollutants, especially Cr, Ni, and Zn, should be regulated or updated in China. Moreover, research on effects of different sources of trace elements on soil contamination and agricultural food safety should be strengthen.

## Supporting information

S1 Supporting Information Table A-Table O. (DOCX)
