# Supplementary material for: Current inventory and changes of the input/output balance of trace elements in farmland across China
Source: PLoS One. 2018 Jun 25;13(6):e0199460. doi: 10.1371/journal.pone.0199460 (PMC6016942; doi:10.1371/journal.pone.0199460)
Supplement: S1 Supporting Information — (DOCX) [file pone.0199460.s001.docx]

List of Tables

[**Table A. Atmospheric deposition flux of trace elements in China and oversea countries or districts (mg/m^2^/year)** 2](#_Toc516929618)

[**Table B. Agricultural utilization ratio of livestock manures in China** 4](#_Toc516929619)

[**Table C. Excretion parameters (fresh weight) and population of livestock in China** 4](#_Toc516929620)

[**Table D. Water contents in livestock manures in China** 4](#_Toc516929621)

[**Table E. The concentrations of trace elements in livestock manures in China (mg/kg)** 5](#_Toc516929622)

[**Table F. The concentrations of trace elements in fertilizers in China (mg/kg)** 6](#_Toc516929623)

[**Table G. The concentrations of trace elements in irrigation water in China (μg/L)** 7](#_Toc516929624)

[**Table H. The concentrations (mg/kg) of trace elements in grains in China** 7](#_Toc516929625)

[**Table I. The concentrations (mg/kg) of trace elements in vegetables in China** 7](#_Toc516929626)

[**Table J. Trace element concentrations (mg/kg) in fruits in China** 7](#_Toc516929627)

[**Table K. Trace element concentrations (mg/kg) in tea in China** 8](#_Toc516929628)

[**Table L. Trace element concentrations (mg/kg) in tobaccos in China** 8](#_Toc516929629)

[**Table M. Trace element concentrations (mg/kg) in oil-bearing crops in China** 8](#_Toc516929630)

[**Table N. Straw/Grain ratio and transfer factors of trace elements in food crops in China** 8](#_Toc516929631)

[**Table O. The trace element outputs from farmland annually in China (t/year)** 9](#_Toc516929632)

**Table A. Atmospheric deposition flux of trace elements in China and oversea countries or districts (mg/m^2^/year)**

| Countries | Study areas | As | Cd | Cr | Cu | Hg | Ni | Pb | Zn |
| --- | --- | --- | --- | --- | --- | --- | --- | --- | --- |
| China (2006-2015) | Number of samples | 755 | 762 | 763 | 764 | 750 | 376 | 768 | 766 |
|  | Ranges | 0.32-9.14 | 0.01-1.42 | 0.61-51.4 | 2.11-45.30 | 0.002-0.860 | 0.53-47.58 | 0.67-66.24 | 10.96-474.60 |
|  | Average±SD | 3.37±2.27 | 0.41±0.36 | 16.14±11.58 | 11.56±8.60 | 0.092±0.186 | 8.08±9.89 | 19.19±13.89 | 72.90±90.61 |
| China (1999-2006) | China (1) | 2.8 | 0.40 | 6.1 | 10.8 | 0.14 | 5.8 | 20.2 | 64.7 |
| America | Massachusetts Bay (2) | 0.022 | 0.27 | 2.7 | 2.5 |  | 1.5 | 1.8 | 7.8 |
|  | Lake Superior (3) | 0.169 | 0.458 | 0.208 | 3.1 |  | 0.8 | 1.47 | 8.8 |
|  | Lake Michigan (3) | 0.138 | 0.452 | 0.202 | 1.87 |  | 0.61 | 1.59 | 6 |
|  | Lake Erie (3) | 0.182 | 0.494 | 1.063 | 4.15 |  | 0.74 | 1.78 | 16.5 |
| England and Wales | England and Wales (4) | 0.31 | 0.19 | 0.75 | 5.7 | 0.1 | 1.6 | 5.4 | 22.1 |
| France | France (5) | 0.053 | 0.025 | 0.239 | 0.802 | 0.008 | 0.298 | 0.772 | 5.568 |
|  | Marais Vernier (6) |  | 0.009 |  |  | 0.009 | 0.362 | 0.142 | 2.98 |
|  | Northern France (7) |  | 0.047 |  | 3.9 |  | 1.5 | 2.2 | 15.6 |
|  | Le Havre (8) |  | 0.36 |  | 9.4 |  | 4.1 | 18.4 | 1172 |
|  | NDG (8) |  | 0.23 |  | 5.2 |  | 2 | 4.8 | 59 |
|  | Rouen (8) |  | 0.19 |  | 2.8 |  | 0.44 | 3 | 27 |
|  | Evreus (8) |  | 0.2 |  | 7.5 |  | 0.34 | 1.7 | 38 |
|  | Paris (8) |  | 0.24 |  | 6 |  | 0.62 | 4.2 | 30 |
| Spain | Doñana National Park (9) | 0.1 | 0.01 | 0.9 | 6.9 |  | 0.3 | 0.7 |  |
|  | Bárcena Mayor (10) | 0.20 | 0.09 | 1.3 | 7.5 |  | 1.2 | 2.5 | 35.3 |
|  | Northern Spain (11) | 0.11 | 0.04 | 1.72 | 4.09 |  | 0.55 | 1.61 | 63.18 |
| New Zealand | Fiorland (12) |  | 0.0044 |  | 0.023 |  |  | 0.035 | 0.025 |
|  | Seven rural sites (13) |  | 0.02 | 2.78 | 3.54 |  | 0.95 | 2.3 | 102.5 |
| Croacia | Kornaty National Park (14) |  | 0.44 |  |  |  |  | 4 |  |
| Sweden | Sweden (4) |  | 0.08 | 0.5 | 1.5 | 0.01 | 0.05 | 0.63 | 11.8 |
| Denmark | North Zealand (15) | 0.13 | 0.04 | 0.3 | 1.4 |  | 0.27 | 0.9 | 7.7 |
|  | Denmark (4) |  | 0.03 | 0.13 | 0.8 |  | 0.21 | 1.04 | 8 |
|  | Southern Scandinavia (16) | 0.1 | 0.05 |  | 1 |  | 0.3 | 1 | 6.9 |
| Finland | Finland (4) | 0.11 | 0.02 | 0.05 | 0.5 |  | 0.15 | 0.57 | 2 |
|  | Virolahti, Finland (17) | 0.24 | 0.1 | 0.49 | 2.7 |  | 0.38 | 3 | 10.4 |
|  | Pallas, Finland (17) | 0.04 | 0.03 | 0.12 | 1.6 |  | 0.15 | 0.49 | 2.5 |
| Lithuania | Aukštaitija IMS (18) | 0.15 | 0.08 | 0.24 | 1.32 | 0.008 | 0.42 | 2.02 | 8.39 |
|  | Žemaitija IMS (18) | 0.34 | 0.21 | 0.46 | 5.98 | 0.014 | 2.60 | 8.08 | 37.32 |
| Serbia | Belgrade (19) |  | 0.22 | 1.64 | 34.5 |  | 11.3 | 21.7 | 41.4 |
| India | Adalpura (20) |  | 0.078 | 0.048 | 0.225 |  | 0.031 | 0.36 | 15.27 |
| Austria | Austria (4) |  | 0.27 | 0.62 | 10 |  | 0.21 | 0.85 | 50 |
| Belgium | Belgium (4) |  |  |  | 0.7 |  | 0.25 | 2.4 | 6.2 |
| Germany | Germany (4) | 0.07 | 0.25 | 0.7 | 5.3 |  | 1.1 | 5.72 | 54 |
| Hungary | Hungary (4) |  | 0.99 |  | 6.2 |  | 2.47 | 10.19 | 21.9 |
| Ireland | Ireland (4) |  | 0.06 | 0.07 | 1.3 |  | 0.16 | 1.33 | 23.5 |
| Italy | Italy (4) |  | 0.33 | 4.56 | 6 |  | 3.5 | 5.81 | 28.9 |
| Norway | Norway (4) |  | 0.06 | 0.17 | 1.2 |  | 0.6 | 1.61 | 6.8 |
| Poland | Poland (4) |  | 0.2 | 3 | 4 |  | 2 | 10 | 54 |
| Switzerland | Switzerland (4) |  | 0.08 | 0.37 | 1.8 |  | 1.1 | 2.8 | 11.9 |
| The Netherlands | The Netherlands (4) | 0.32 | 0.13 | 0.25 | 2.7 | 0.07 | 1.06 | 4.7 | 16.2 |
| Japan | Kushiro (21) |  | 0.06 |  | 1.6 |  | 2 | 2.7 | 11 |
|  | Otsuchi (21) |  | 0.14 |  | 6.1 |  | 2 | 9 | 40 |
|  | Hedo (21) |  | 0.21 |  | 0.96 |  | 2.2 | 5.9 | 15 |
|  | Tokyo Bay (22) | 2.9 | 0.39 | 6.2 | 16 | 0.04 | 6.8 | 9.9 |  |
| North Sea | North Sea (23) | 0.25 |  | 2.43 | 1.3 |  | 1.21 | 3.69 | 6.5 |
| Overseas average | | 0.30 | 0.18 | 1.14 | 4.46 | 0.03 | 1.41 | 3.88 | 50.24 |

Notes: Principles of atmospheric deposition data collection and processing are as follows: (1) In order to reflect the current situation of atmospheric deposition fluxes of trace elements in China, the data from publication that samples were collected during 2006-2015 were selected. (2) Atmospheric deposition samples collected in cities, industrial or mining areas were removed from database, and samples from non-contaminated areas like agricultural or background areas were retained, in order to avoid possible interference from various pollution sources. (3) Sampling period of atmospheric deposition was at least one year or more. (4) A geometric mean was calculated as a representative if there was more data in a selected area. (5) Atmospheric deposition fluxes of trace elements in China were varied greatly in China, therefore the ratio of agricultural soil area of a selected province to the total agricultural soil area in China was selected as weighted value, and then atmospheric deposition fluxes of trace elements in the whole country were calculated using a weighting method.

**Table B. Agricultural utilization ratio of livestock manures in China**

| No. | Research area | Utilization ratio | References |
| --- | --- | --- | --- |
| 1 | Yancheng city, Jiangsu province | 40% | (24) |
| 2 | Jiangyan district, Jiangsu province | 40% | (25) |
| 3 | Guannan county, Jiangsu province | 42% | (26) |
| 4 | Yancheng city, Jiangsu province | 31% | (27) |
| 5 | Liaozhong district, Liaoning province | 56% | (28) |
| 6 | Inner Mongolia, Liaoning, Jilin and Heilongjiang provinces | 47% | (29) |
|  | Geometric mean | 42% |  |

**Table C. Excretion parameters (fresh weight) and population of livestock in China**

| Livestock | Excretion parameters ^1^ | Population of livestock (10^4^ heads) | |
| --- | --- | --- | --- |
|  |  | 2005^2^ | 2015^3^ |
| Hogs | 5.3 kg/day | 66098.6 | 70825 |
| Draft cow | 10.1 t/year | 7190 | 1937.2 |
| Beeves | 7.7 t/year | 5750 | 5003.4 |
| Dairy cow | 19.4 t/year | 1216.1 | 1507.2 |
| Horse | 5.9 t/year | 740.0 | 590.8 |
| Donkey and mule | 5 t/year | 1137.6 | 752.1 |
| Sheep | 0.87 t/year | 37265.9 | 31099.7 |
| Dorking | 0.1 kg/day | 217720 | 904400 |
| Layer | 53.3 kg/year | 566450 | 143400 |
| Duck and goose | 39 kg/year | 202420 | 294600 |

Notes: ^1^ Wang et al. (2006) (30); ^2^ Luo et al. (2009) (1, 31); ^3^ China Agriculture Statistical Yearbook 2016 (32).

**Table D. Water contents in livestock manures in China**

| Livestock | Water content | References |
| --- | --- | --- |
| Hog | 73% | (33) |
| Cow | 85% | (33) |
| Sheep | 70% | (34, 35) |
| Poultry | 75% | (33) |

**Table E. The concentrations of trace elements in livestock manures in China (mg/kg)**

|  |  | **As** | **Cd** | **Cr** | **Cu** | **Hg** | **Ni** | **Pb** | **Zn** |
| --- | --- | --- | --- | --- | --- | --- | --- | --- | --- |
| Poultry | Number | 322 | 378 | 344 | 381 | 265 | 161 | 357 | 363 |
|  | Ranges | n.d.-82.30 | n.d.-63.64 | 0.34-4304.68 | 1.53-1274.00 | n.d.-1.34 | 0.93-27.57 | n.d.-150.50 | 15.37-4485.00 |
|  | GeoMean | 3.81 | 0.5 | 25.17 | 76.4 | 0.07 | 7.08 | 7.65 | 289.97 |
|  | SD | 20.23 | 11.57 | 870 | 298.1 | 0.38 | 7.68 | 35.09 | 820.09 |
| Duck | Number | 48 | 66 | 66 | 66 | 38 | 38 | 48 | 66 |
|  | Ranges | 0.62-18.10 | 0.04-3.85 | 4.49-421.76 | 18.56-835.30 | 0.03-0.12 | 5.34-15.43 | 4.00-80.61 | 63.89-7317.61 |
|  | GeoMean | 4.46 | 0.42 | 38.17 | 47.07 | 0.06 | 8.72 | 12.62 | 292.07 |
|  | SD | 6.11 | 0.83 | 79.4 | 163.87 | 0.04 | 4.71 | 25.25 | 1383.76 |
| Sheep | Number | 14 | 36 | 14 | 27 | 14 | 13 | 24 | 15 |
|  | Ranges | 0.20-8.23 | 0.06-8.39 | 0.40-72.30 | 2.67-510.50 | 0.01-11.55 | 0.57-6.87 | 0.36-21.90 | 0.12-676.65 |
|  | GeoMean | 0.87 | 0.59 | 5.2 | 23.91 | 0.1 | 2.93 | 3.76 | 36.49 |
|  | SD | 3.42 | 2.85 | 30.44 | 185.68 | 5.14 | 3 | 8.17 | 276.27 |
| Cattle | Number | 257 | 358 | 281 | 364 | 179 | 122 | 300 | 293 |
|  | Ranges | n.d.-69.50 | n.d.-35.50 | n.d.-678.70 | 5.58-1257.13 | n.d.-2.54 | 0.37-21.69 | 0.01-117.00 | 12.46-2713.00 |
|  | GeoMean | 2.43 | 0.4 | 12.01 | 55.85 | 0.04 | 6.62 | 6.06 | 162.23 |
|  | SD | 13.21 | 6.33 | 127.51 | 254.01 | 0.57 | 7.18 | 24.27 | 615.7 |
| Swine | Number | 770 | 1225 | 980 | 1244 | 589 | 275 | 1097 | 1124 |
|  | Range | n.d.-373.80 | n.d.-203.40 | n.d.-1616.52 | 5.91-2030.00 | n.d.-9.42 | 2.14-559.10 | n.d.-825.20 | 1.53-14679.80 |
|  | GeoMean | 8.53 | 1.06 | 13.74 | 298.98 | 0.06 | 10.48 | 5.62 | 712.13 |
|  | SD | 74.9 | 30.85 | 261.89 | 583.48 | 2.19 | 109.13 | 110.63 | 3312.2 |

Notes: GeoMean, geometric mean; SD, standard deviation.

**Table F. The concentrations of trace elements in fertilizers in China (mg/kg)**

| Fertilizers |  | As | Cd | Cr | Cu | Hg | Ni | Pb | Zn | Amount of fertilizers applied (10^4^ tons) | |
| --- | --- | --- | --- | --- | --- | --- | --- | --- | --- | --- | --- |
|  |  |  |  |  |  |  |  |  |  | 2005^1^ | 2015^2^ |
| Nitrogen | Number | 54 | 65 | 53 | 58 | 60 | 36 | 64 | 58 | 2229.3 | 2361.6 |
|  | Ranges | 0.01-7.25 | n.d.-0.674 | n.d.-73.490 | n.d.-49.960 | n.d.-0.911 | n.d.-34.11 | n.d.-8.61 | n.d.-49.19 |  |  |
|  | GeoMean | 0.145 | 0.041 | 1.936 | 1.631 | 0.03 | 14.63 | 1.08 | 3.01 |  |  |
|  | SD | 0.182 | 0.021 | 1.913 | 1.067 | 0.032 | 1.83 | 0.31 | 1.07 |  |  |
| Phosphate | Number | 59 | 72 | 48 | 36 | 53 | 23 | 66 | 36 | 743.8 | 843.1 |
|  | Ranges | n.d.-51.70 | n.d.-27.2 | 0.10-751.60 | n.d.-556.10 | n.d.-41.30 | 0.05-2518.83 | n.d.-234.10 | n.d.-1323.60 |  |  |
|  | GeoMean | 4.52 | 0.49 | 38.6 | 17.38 | 0.111 | 22.05 | 11.35 | 51.3 |  |  |
|  | SD | 1.61 | 0.49 | 20.68 | 18.29 | 0.788 | 109.99 | 5.39 | 36.21 |  |  |
| Potash | Number | 18 | 23 | 20 | 14 | 19 | 11 | 22 | 14 | 489.5 | 642.3 |
|  | Ranges | 0.01-12.47 | n.d.-5.25 | n.d.-98.21 | 0.03-34.69 | n.d.-10.04 | 0.04-65.54 | 0.04-99.30 | 0.25-104.06 |  |  |
|  | GeoMmean | 0.35 | 0.09 | 2.39 | 2.8 | 0.03 | 4.21 | 3.42 | 5.15 |  |  |
|  | SD | 0.88 | 0.24 | 6 | 2.69 | 0.52 | 6.89 | 6.51 | 9.3 |  |  |
| Compound | Number | 113 | 137 | 109 | 92 | 101 | 57 | 134 | 100 | 1303.2 | 2175.7 |
|  | Ranges | n.d.-126.54 | n.d.-63.81 | n.d.-232.00 | n.d.-869.90 | n.d.-6.30 | 1.80-199.36 | n.d.-89.78 | 1.44-2588.41 |  |  |
|  | GeoMean | 2.98 | 0.49 | 10.65 | 13.86 | 0.037 | 25.53 | 3.09 | 43.78 |  |  |
|  | SD | 1.72 | 0.78 | 3.33 | 10.44 | 0.096 | 3.47 | 1.46 | 33.07 |  |  |

Notes: ^1^ China Statistical Yearbook 2006 (36); ^2^ China Statistical Yearbook 2016 (37); GeoMean, geometric mean; SD, standard deviation.

**Table G. The concentrations of trace elements in irrigation water in China (μg/L)**

|  | As | Cd | Cr | Cu | Hg | Ni | Pb | Zn |
| --- | --- | --- | --- | --- | --- | --- | --- | --- |
| Number | 3268 | 3540 | 1712 | 3397 | 1313 | 1896 | 2563 | 3195 |
| Ranges | n.d.-5383.3 | n.d.-269.0 | n.d.-3591.0 | n.d.-1210.0 | n.d.-894.3 | n.d.-310.0 | n.d.-1239.9 | n.d.-50700.0 |
| GeoMean | 1.34 | 0.13 | 2.41 | 4.86 | 0.037 | 1.84 | 2.01 | 13.53 |
| SD | 362.78 | 20.99 | 143.86 | 62.79 | 67.424 | 31.11 | 173.79 | 1868.88 |

Notes: GeoMean, geometric mean; SD, standard deviation.

**Table H. The concentrations (mg/kg) of trace elements in grains in China**

|  |  | As | Cd | Cr | Cu | Hg | Ni | Pb | Zn |
| --- | --- | --- | --- | --- | --- | --- | --- | --- | --- |
| Total grain | Number | 5826 | 12282 | 1703 | 1789 | 6877 | 1066 | 9223 | 1561 |
|  | Ranges | n.d.-5.70 | n.d.-7.08 | n.d.-25.31 | n.d.-16.20 | n.d.-2.310 | n.d.-4.71 | n.d.-10.29 | 0.03-175.52 |
|  | GeoMean | 0.04 | 0.016 | 0.3 | 2.87 | 0.003 | 0.32 | 0.04 | 21.38 |
|  | SD | 0.60 | 0.382 | 2.81 | 2.65 | 0.170 | 0.81 | 0.82 | 18.1 |
| Rice grain | Number | 3683 | 7873 | 682 | 599 | 4005 | 166 | 5167 | 438 |
|  | Ranges | n.d.-5.70 | n.d.-7.08 | n.d.-25.31 | 0.20-9.80 | n.d.-1.060 | 0.040-1.820 | n.d.-10.29 | 5.29-44.30 |
|  | GeoMean | 0.1 | 0.02 | 0.3 | 3.73 | 0.003 | 0.36 | 0.05 | 17.87 |
|  | SD | 0.77 | 0.51 | 3.95 | 2.44 | 0.108 | 0.39 | 0.97 | 7.49 |
| Wheat grain | Number | 578 | 1610 | 552 | 563 | 532 | 470 | 1052 | 568 |
|  | Ranges | n.d.-3.24 | n.d.-1.09 | n.d.-11.21 | n.d.-11.30 | n.d.-0.230 | n.d.-4.41 | n.d.-3.92 | 0.03-175.52 |
|  | GeoMean | 0.04 | 0.02 | 0.48 | 3.33 | 0.002 | 0.28 | 0.04 | 21.78 |
|  | SD | 0.67 | 0.14 | 2.59 | 2.39 | 0.034 | 1.022 | 0.63 | 30.97 |
| Maize grain | Number | 504 | 623 | 395 | 525 | 389 | 430 | 650 | 549 |
|  | Ranges | n.d.-1.33 | n.d.-0.310 | n.d.-3.24 | 0.22-8.03 | n.d.-0.197 | n.d.-4.71 | n.d.-2.63 | 0.21-68.85 |
|  | GeoMean | 0.02 | 0.014 | 0.24 | 1.9 | 0.003 | 0.25 | 0.04 | 25.65 |
|  | SD | 0.24 | 0.06 | 0.60 | 1.52 | 0.034 | 1.27 | 0.45 | 10.70 |

Notes: GeoMean, geometric mean; SD, standard deviation.

**Table I. The concentrations (mg/kg) of trace elements in vegetables in China**

|  | As | Cd | Cr | Cu | Hg | Ni | Pb | Zn |
| --- | --- | --- | --- | --- | --- | --- | --- | --- |
| Number | 6240 | 8504 | 4091 | 1682 | 7582 | 906 | 9401 | 1707 |
| Ranges | n.d.-18.120 | n.d.-26.620 | n.d.-84.240 | n.d.-192.560 | n.d.-10.200 | n.d.-4.600 | n.d.-241.300 | n.d.-549.03 |
| GeoMean | 0.017 | 0.019 | 0.076 | 1.025 | 0.003 | 0.02 | 0.056 | 6.139 |
| SD | 1.423 | 1.841 | 4.440 | 19.404 | 0.605 | 0.669 | 14.508 | 58.356 |

Notes: GeoMean, geometric mean; SD, standard deviation.

**Table J. Trace element concentrations (mg/kg) in fruits in China**

|  | As | Cd | Cr | Cu | Hg | Ni | Pb | Zn |
| --- | --- | --- | --- | --- | --- | --- | --- | --- |
| Number | 979 | 1366 | 307 | 144 | 859 | 242 | 1767 | 143 |
| Ranges | n.d.-4.061 | n.d.-0.187 | n.d.-3.013 | 0.171-3.448 | n.d.-0.155 | n.d.-0.712 | n.d.-0.355 | 0.282-4.584 |
| GeoMean | 0.012 | 0.003 | 0.172 | 0.541 | 0.003 | 0.058 | 0.019 | 1.121 |
| SD | 0.547 | 0.026 | 0.566 | 0.689 | 0.038 | 0.155 | 0.073 | 1.237 |

Notes: GeoMean, geometric mean; SD, standard deviation.

**Table K. Trace element concentrations (mg/kg) in tea in China**

|  | As | Cd | Cr | Cu | Hg | Ni | Pb | Zn |
| --- | --- | --- | --- | --- | --- | --- | --- | --- |
| Number | 355 | 570 | 996 | 434 | 185 | 355 | 1645 | 395 |
| Ranges | n.d.-1.58 | n.d.-3.79 | n.d.-21.37 | 0.01-44.07 | n.d.-0.303 | 0.53-15.58 | n.d.-30.75 | 3.59-201.70 |
| GeoMean | 0.06 | 0.04 | 0.39 | 6.17 | 0.015 | 3.42 | 0.66 | 30.57 |
| SD | 0.27 | 0.55 | 3.01 | 11.65 | 0.106 | 2.69 | 2.85 | 32.53 |

Notes: GeoMean, geometric mean; SD, standard deviation.

**Table L. Trace element concentrations (mg/kg) in tobaccos in China**

|  | As | Cd | Cr | Cu | Hg | Ni | Pb | Zn |
| --- | --- | --- | --- | --- | --- | --- | --- | --- |
| Number | 2245 | 2177 | 2109 | 550 | 2103 | 556 | 2273 | 397 |
| Ranges | 0.03-20.60 | 0.07-25.80 | n.d.-24.27 | 0.94-50.64 | n.d.-3.000 | 0.31-16.09 | 0.17-16.95 | 0.27-462.05 |
| GeoMean | 0.46 | 2.58 | 1.14 | 8.58 | 0.052 | 1.88 | 3.33 | 20.69 |
| SD | 1.80 | 5.07 | 2.85 | 7.49 | 0.246 | 2.31 | 2.95 | 51.94 |

Notes: GeoMean, geometric mean; SD, standard deviation.

**Table M. Trace element concentrations (mg/kg) in oil-bearing crops in China**

|  | As | Cd | Cr | Cu | Hg | Ni | Pb | Zn |
| --- | --- | --- | --- | --- | --- | --- | --- | --- |
| Number | 95 | 182 | 82 | 59 | 68 | 41 | 150 | 94 |
| Ranges | n.d.-0.40 | n.d.-0.68 | n.d.-2.14 | 2.15-16.07 | n.d.-0.100 | 0.17-23.00 | n.d.-9.95 | 1.20-68.10 |
| GeoMean | 0.03 | 0.07 | 0.19 | 8.72 | 0.003 | 1.60 | 0.02 | 35.41 |
| SD | 0.08 | 0.14 | 0.55 | 3.08 | 0.021 | 7.11 | 1.55 | 13.06 |

Notes: GeoMean, geometric mean; SD, standard deviation.

**Table N. Straw/Grain ratio and transfer factors of trace elements in food crops in China**

|  |  | S/G ratio^1^ | Transfer factors^2^ | | | | | | | |
| --- | --- | --- | --- | --- | --- | --- | --- | --- | --- | --- |
|  |  |  | As | Cd | Cr | Cu | Hg | Ni | Pb | Zn |
| Rice | Number | 125 | 62 | 71 | 24 | 56 | 20 | 32 | 46 | 32 |
|  | Ranges | 0.4-32.0 | 4.0-80.4 | 0.4-46.0 | 0.5-52.9 | 0.3-28.2 | 1.3-15.2 | 1.6-54.1 | 0.7-27.8 | 0.7-4.5 |
|  | Geomean^3^ | 1.3 | 23.3 | 3.1 | 4.3 | 1.6 | 6.0 | 9.5 | 5.2 | 1.8 |
|  | SD | 0.3 | 2.3 | 1.2 | 3.4 | 0.6 | 1.1 | 2.9 | 1.0 | 0.2 |
| Wheat | Number | 167 | 35 | 67 | 56 | 69 | 45 | 25 | 53 | 68 |
|  | Ranges | 0.5-3.9 | 1.3-24.9 | 1.0-67.9 | 0.1-122.0 | 0.3-3.0 | 1.0-121.6 | 0.1-133.0 | 2.4-60.9 | 0.2-1.6 |
|  | Geomean | 1.3 | 6.5 | 3.8 | 5.4 | 0.8 | 6.7 | 3.5 | 12.4 | 0.5 |
|  | SD | 0.05 | 1.0 | 1.6 | 3.2 | 0.1 | 4.0 | 9.4 | 2.1 | 0.02 |
| Corn | Number | 94 | 16 | 45 | 29 | 37 | 4 | 26 | 40 | 37 |
|  | Ranges | 0.4-6.9 | 3.7-31.1 | 1.3-110.0 | 0.6-246.7 | 0.4-9.6 | 1.0-3.5 | 1.5-12.7 | 0.4-148.0 | 0.3-5.3 |
|  | Geomean | 1.1 | 11.9 | 6.9 | 11.5 | 2.8 | 2.2 | 5.2 | 6.7 | 1.3 |
|  | SD | 0.1 | 2.6 | 4.1 | 9.9 | 0.4 | 0.6 | 0.5 | 5.3 | 0.2 |

Notes: ^1^ weight ratio of straw/grain; ^2^ Ratio of trace element concentrations in straw/grain; GeoMean, geometric mean; SD, standard deviation.

**Table O. The trace element outputs from farmland annually in China (t/year)**

|  |  | As | Cd | Cr | Cu | Hg | Ni | Pb | Zn |
| --- | --- | --- | --- | --- | --- | --- | --- | --- | --- |
| Crop  harvesting | Rice | 20.82 | 4.16 | 62.47 | 776.68 | 0.62 | 74.96 | 10.41 | 3720.98 |
|  | Wheat | 5.21 | 2.60 | 62.49 | 433.52 | 0.26 | 36.45 | 5.21 | 2835.43 |
|  | Maize | 4.49 | 3.14 | 53.91 | 426.80 | 0.67 | 56.16 | 8.99 | 5761.81 |
|  | Other cereal | 2.34 | 0.93 | 17.52 | 167.60 | 0.18 | 18.69 | 2.34 | 1248.53 |
|  | Oil-bearing crops | 1.06 | 2.48 | 6.72 | 308.43 | 0.11 | 56.59 | 0.71 | 1252.45 |
|  | Cotton | 0.17 | 0.17 | 0.62 | 7.40 |  | 4.93 | 2.58 |  |
|  | Sugarcane |  | 3.39 | 45.62 |  |  |  | 13.22 |  |
|  | Sugarbeet |  |  |  | 150.20 |  |  |  | 471.48 |
|  | Tobacco | 1.30 | 7.31 | 3.23 | 24.30 | 0.15 | 5.32 | 9.43 | 58.59 |
|  | Tea | 0.13 | 0.09 | 0.88 | 13.88 | 0.03 | 7.69 | 1.48 | 68.75 |
|  | Fruit | 3.29 | 0.82 | 47.09 | 148.10 | 0.82 | 15.88 | 5.20 | 306.87 |
|  | Vegetable | 13.35 | 14.92 | 59.68 | 804.89 | 2.36 | 15.71 | 43.97 | 4820.72 |
| Straw removing | Rice | 201.20 | 5.35 | 111.39 | 515.34 | 1.55 | 295.32 | 22.45 | 2777.57 |
|  | Wheat | 14.04 | 4.10 | 139.94 | 143.82 | 0.72 | 52.91 | 26.78 | 587.93 |
|  | Maize | 18.76 | 7.61 | 217.55 | 419.34 | 0.52 | 102.47 | 21.12 | 2628.35 |
|  | Crop harvesting | 52.17 | 40.02 | 360.22 | 3261.78 | 5.20 | 292.38 | 103.53 | 20545.62 |
|  | Straw removing | 233.99 | 17.07 | 468.88 | 1078.51 | 2.80 | 450.70 | 70.35 | 5993.85 |
|  | Total output | 286.16 | 57.09 | 829.10 | 4340.29 | 8.00 | 743.08 | 173.89 | 26539.46 |

Notes: Trace element concentrations in other grain were treated as averages of trace element in total grain, and yield of other grain was calculated as: Yield of other grain = Yield of total grain – yield of main food grain (rice, wheat, and maize). Trace element concentrations in sugarcane and beet were from Dao et al. (2015) (38) and Jia et a. (2015) (39), respectively. Trace element concentrations in cotton were from Qiao et al. (2016) (40).

**References**

1. Luo L, Ma Y, Zhang S, Wei D, Zhu Y. An inventory of trace element inputs to agricultural soils in China. Journal of Environmental Management. 2009;90(8):2524-2530.

2. Golomb D, Ryan D, Eby N, Underhill J, Zemba S. Atmospheric deposition of toxics onto Massachusetts Bay—I. Metals. Atmospheric Environment. 1997;31(9):1349-1359.

3. Sweet CW, Weiss A, Vermette SJ. Atmospheric deposition of trace metals at three sites near the Great Lakes. Water, Air, and Soil Pollution. 1998;103(1):423-439.

4. Nicholson FA, Smith SR, Alloway BJ, Carlton-Smith C, Chambers BJ. An inventory of heavy metals inputs to agricultural soils in England and Wales. Science of the total environment. 2003;311(1):205-219.

5. Belon E, Boisson M, Deportes IZ, Eglin TK, Feix I, Bispo AO, et al. An inventory of trace elements inputs to French agricultural soils. Science of the Total Environment. 2012;439C(22):87-95.

6. Connan O, Maro D, Hébert D, Roupsard P, Goujon R, Letellier B, et al. Wet and dry deposition of particles associated metals (Cd, Pb, Zn, Ni, Hg) in a rural wetland site, Marais Vernier, France. Atmospheric Environment. 2013;67(Supplement C):394-403.

7. Azimi S, Cambier P, Lecuyer I, Thevenot D. Heavy Metal Determination in atmospheric deposition and other fluxes in northern France agrosystems. Water, Air, and Soil Pollution. 2004;157(1):295-313.

8. Motelay-Massei A, Ollivon D, Tiphagne K, Garban B. Atmospheric bulk deposition of trace metals to the Seine River Basin, France: concentrations, sources and evolution from 1988 to 2001 in Paris. Water, Air, and Soil Pollution. 2005;164(1):119-135.

9. Castillo S, de la Rosa JD, Sánchez de la Campa AM, González-Castanedo Y, Fernández-Camacho R. Heavy metal deposition fluxes affecting an Atlantic coastal area in the southwest of Spain. Atmospheric Environment. 2013;77(Supplement C):509-517.

10. Fernández-Olmo I, Puente M, Irabien A. A comparative study between the fluxes of trace elements in bulk atmospheric deposition at industrial, urban, traffic, and rural sites. Environmental Science and Pollution Research. 2015;22(17):13427-13441.

11. Fernández-Olmo I, Puente M, Montecalvo L, Irabien A. Source contribution to the bulk atmospheric deposition of minor and trace elements in a Northern Spanish coastal urban area. Atmospheric Research. 2014;145-146(Supplement C):80-91.

12. Halstead MJR, Cunninghame RG, Hunter KA. Wet deposition of trace metals to a remote site in Fiordland, New Zealand. Atmospheric Environment. 2000;34(4):665-676.

13. Gray CW, McLaren RG, Roberts AHC. Atmospheric accessions of heavy metals to some New Zealand pastoral soils. Science of The Total Environment. 2003;305(1):105-115.

14. Čačković M, Kalinić N, Vadjić V, Pehnec G. Heavy metals and acidic components in total deposited matter in Šibenik and National Park Kornati, Croatia. Archives of Environmental Contamination and Toxicology. 2009;56(1):12-20.

15. Hovmand MF, Kystol J. Atmospheric element deposition in southern Scandinavia. Atmospheric Environment. 2013;77(Supplement C):482-489.

16. Hovmand MF, Kemp K, Kystol J, Johnsen I, Riis-Nielsen T, Pacyna JM. Atmospheric heavy metal deposition accumulated in rural forest soils of southern Scandinavia. Environmental Pollution. 2008;155(3):537-541.

17. Kyllönen K, Karlsson V, Ruoho-Airola T. Trace element deposition and trends during a ten year period in Finland. Science of The Total Environment. 2009;407(7):2260-2269.

18. Kvietkus K, Šakalys J, Valiulis D. Trends of atmospheric heavy metal deposition in Lithuania. Lithuanian Journal of Physics. 2011;51(4):359-369.

19. Tasić M, Mijić Z, Rajšić S, Stojić A, Radenković M, Joksić J, editors. Source apportionment of atmospheric bulk deposition in the Belgrade urban area using Positive Matrix factorization2009.

20. Pandey J, Shubhuashish K, Pandey R. Heavy metal contamination of Ganga river at Varanasi in relation to atmospheric deposition. Tropical Ecology. 2010;51(2S):365-373.

21. Okubo A, Takeda S, Obata H. Atmospheric deposition of trace metals to the western North Pacific Ocean observed at coastal station in Japan. Atmospheric Research. 2013;129-130(Supplement C):20-32.

22. Sakata M, Tani Y, Takagi T. Wet and dry deposition fluxes of trace elements in Tokyo Bay. Atmospheric Environment. 2008;42(23):5913-5922.

23. Injuk J, Grieken RV, Leeuw GD. Deposition of atmospheric trace elements into the north sea: Coastal, ship, platform measurements and model predictions. Atmospheric Environment. 1998;32(17):3011-3025.

24. Tan G. Current situation of livestock manures disposal and utilization in Jiangsu province. Livestock and Poultry Industry. 2014(301):58-60.

25. Liu H, Li S, Wu F. Current situation of livestock manures disposal and utilization in Jiangyan city. Modern Agricultural Science and Technology. 2012(21):244-245.

26. Piao C. Current situation of livestock manures disposal and utilization in Guannan county. The Chinese Livestock and Poultry Breeding. 2014(1):19-20.

27. Zuo Q, Xu H. Current situation of livestock manures disposal and utilization in Yandu district, Yancheng city. Modern Agricultural Science and Technology. 2011(16):262.

28. Lv J, Wang Z, Xi F, Bing L, Wang M. Present Situation, Potential and countermeasures of Livestock Manure Recycling in the Circular Agriculture: A Case in Liaozhong County. Ecological Economy. 2015;31(4):107-113.

29. Yang F, Dong Y. Livestock manures organic fertilizers resources in four provinces in northeast China and its utilization. China Agricultural Technology Extension. 2015;31(1):40-41.

30. Wang F, Ma W, Dou Z, Ma L, Liu x, Xu J, et al. The estimation of the production amount of animal manure and its environmental effect in China. China Environmental Sciences. 2006;25(5):614-617.

31. Ministry of Agriculture of the People's Republic of China. China Agriculture Statistical Yearbook 2006. Beijing: China Agriculture Press; 2006.

32. Ministry of Agriculture of the People's Republic of China. China Agriculture Statistical Yearbook 2016. Beijing: China Agriculture Press; 2016.

33. Fei H, Chang Z, Wang S, Huang H, Chen X, Zhu H. Characterization of moisture in three livestock manures. Journal of Agro-Environment Science. 2006;25(Suppl.):599-603.

34. Shi J, Zhou S, Zhao J, Dong B. Livestock and poultry manure excrements and their environmental effect evaluation in Nanyang. Acta Ecologiae Animalis Domastici. 2014;35(12):76-81.

35. Guo D, Wu H, Ma Y, Chang Z. Study on the amount of manure and urine excreted by sheep and rabbits in intensive pasture. Journal of Ecology and Rural Environment. 2011;27(1):44-48.

36. National Bureau of Statistics of China. China Statistical Yearbook 2006. Beijing: China Statistics Press; 2006.

37. National Bureau of Statistics of China. China Statistical Yearbook 2016. Beijing: China Statistics Press; 2016.

38. Dao J, Wu Y, Deng J, Fan x, Zhang F, Fang Z, et al. Risk assessmeng of the content heavy mentals - cadmium, chromium, and lead in sugarcane. Sugar Crops of China. 2015;37(5):10-12.

39. Jia X, Zhu S, Wang Q, Long W, Zhang X. Principal component analysis and cluster analysis of the elements in sugar beet roots of different geographical origin in Xinjiang. Modern Food Science and Technolgy. 2015;31(7):302-308.

40. Qiao Q, Guo H, He B. Determination and safety evaluation of heavy metals in cotton fiber. Journal of Inspection and Quarantine. 2016;26(5):32-34.
